# SARS-CoV-2 Circulation during the First Year of the Pandemic: A Seroprevalence Study from January to December 2020 in Tuscany, Italy

**DOI:** 10.3390/v14071441

**Published:** 2022-06-30

**Authors:** Serena Marchi, Gianvito Lanave, Michele Camero, Francesca Dapporto, Alessandro Manenti, Linda Benincasa, Angela Acciavatti, Giulio Brogi, Simonetta Viviani, Emanuele Montomoli, Claudia Maria Trombetta

**Affiliations:** 1 Department of Molecular and Developmental Medicine, University of Siena, 53100 Siena, Italy; simonetta.viviani@unisi.it (S.V.); emanuele.montomoli@unisi.it (E.M.); trombetta@unisi.it (C.M.T.); 2 Department of Veterinary Medicine, University of Bari, 70010 Valenzano, Italy; gianvito.lanave@uniba.it (G.L.); michele.camero@uniba.it (M.C.); 3 VisMederi Srl, 53100 Siena, Italy; francesca.dapporto@vismederi.com (F.D.); alessandro.manenti@vismederi.com (A.M.); 4 VisMederi Research Srl, 53100 Siena, Italy; linda.benincasa@vismederiresearch.com; 5 NeoMedica Srl, 53100 Siena, Italy; angelaacciavatti@gmail.com (A.A.); laboratorio@neomedicasiena.it (G.B.)

**Keywords:** SARS-CoV-2, Italy, seroprevalence

## Abstract

Italy was the second country affected by the SARS-CoV-2 pandemic; the virus spread mainly in Northern Italy with a subsequent diffusion to the center and southern part of the country. In this study, we aimed to assess the prevalence of antibodies against SARS-CoV-2 in the general population of the Siena province in the Tuscany region (Central Italy) during 2020. A total of 2480 serum samples collected from January to December 2020 were tested for IgM and IgG antibodies against SARS-CoV-2 by a commercial ELISA. Positive and borderline samples were further tested for the presence of anti-receptor-binding domain (RBD) IgM and IgG antibodies by an in-house ELISA and by a micro-neutralization assay. Out of the 2480 samples tested by the commercial ELISA, 81 (3.3%) were found to be positive or borderline for IgG and 58 (2.3%) for IgM in a total of 133 samples (5.4%) found to be positive or borderline for at least one antibody class. When the commercial ELISA and in-house ELISA/micro-neutralization assay results were combined, 26 samples (1.0%) were positive for RBD IgG, 11 (0.4%) for RBD IgM, and 23 (0.9%) for a neutralizing antibody. An increase in seroprevalence was observed during the year 2020, especially from the end of summer, consistent with the routine epidemiological surveillance of COVID-19 cases.

## 1. Introduction

On 11 March 2020, the World Health Organization (WHO) declared the first pandemic caused by a coronavirus. The initial epidemic originated in China, where cases of pneumonia of an unknown etiology were reported in late December 2019. On 7 January 2020, a new coronavirus was isolated and later named Severe Acute Respiratory Syndrome Coronavirus 2 (SARS-CoV-2) by the WHO because the virus was genetically related to the coronavirus responsible for the 2003 SARS outbreak. The new disease caused by SARS-CoV-2 was named COVID-19 (coronavirus disease) [1].

On 22 February 2020, clusters of COVID-19 cases were reported in the Lombardy region, Northern Italy; the transmission was assumed to be local rather than caused by people travelling to or returning from affected areas [2]. The measures of social distancing, aimed at containing the spread of the infection, were initially limited to the affected municipalities of the Lombardy and Veneto regions and were labelled as a “red zone”. The “red zone” was subsequently extended to areas of the Emilia-Romagna, Piedmont, and Marche regions [3]. On 4 March 2020, social containment measures were introduced at a national level and on 9 March a national lockdown (also called “Phase 1”) was declared. The lockdown phase was characterized by the implementation of measures aimed at reducing and preventing the risk of social gatherings and person-to-person interactions such as the closure of non-essential commercial and productive sites, the prohibition of social events and exhibitions, the closure of schools at all levels, the large-scale institution of home-based work, and the limitation of individual mobility [4]. The first pandemic wave, which lasted from the end of February to early May 2020, mainly occurred in the Northern regions, in particular the Lombardy region [5].

Following a decline in morbidity, mortality, and infections, from 4 May 2020, Italy entered “Phase 2”, with the gradual reopening of work, commercial, and recreational activities and the restoration of internal and international travelling. The relaxation of the restrictive measures continued from 15 June, defining the so-called “Phase 3” [3]. This phase lasted until the end of July 2020 and was characterized by a decrease in cases followed by a stabilization within a low incidence context. A slight, but steady, increase in cases occurred, especially from mid-August when the effective reproduction number (Rt) exceeded the threshold of 1 [4], triggering the second pandemic wave that hit Italy throughout the country from the north to the south [5]. New restrictive measures were implemented in October 2020 and became more stringent as the epidemic curve increased. Regions were labelled according to three levels (yellow, orange, and red), which identified the areas with increasing levels of COVID-19 morbidity and mortality. Corresponding levels of social restrictive measures were implemented; further restrictive measures were also applied throughout the national territory until the end of 2020 and the beginning of 2021, a time frame when social mobility is usually high [3].

**Figure 1:**
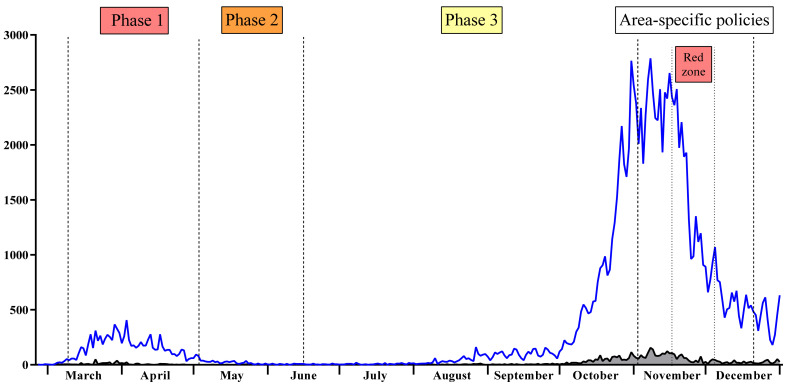
New SARS-CoV-2 infection cases from 24 February to 31 December 2020 in the Tuscany region (blue line) and in the province of Siena (grey line), according to the Italian Department of Civil Protection [6]. Vertical dashed lines indicate the adoption of restrictive measures by time.

During the first epidemic wave, the Tuscany region in Central Italy had a weekly incidence rate of new positive cases per 100,000 inhabitants of 19.4, which was lower than the national average of 28 (Figure 1). These cases mainly occurred in the north-west area of the region (provinces of Massa, Lucca, and Florence). During the second wave, the weekly incidence rate increased to 154 new positive cases per 100,000 inhabitants (the national average was 127) (Figure 1) and other provinces in the region were affected [6,7]. During the second epidemic wave, the province of Siena remained one of the least affected provinces, probably due to its geographical conformation and low population density [6] (Figure 1). The Tuscany region was subject to social restrictions from 11 November to 18 December 2020 and was declared a “red zone” from 13 November to 3 December 2020 (Figure 1).

On the basis of confirmed SARS-CoV-2, asymptomatic and mild-symptomatic infections are far more numerous than severe and fatal cases. For this purpose, seroepidemiological studies have the advantage of providing population data on past exposure to the virus and may help to better determine the true number of infections within the general population [8].

With the purpose of retrospectively evaluating the extent of SARS-CoV-2 circulation during the first year of the pandemic, we assessed the prevalence of antibodies against SARS-CoV-2 in a sample population of the Siena province of the Tuscany region, Central Italy, during 2020.

## 2. Materials and Methods

### 2.1. Study Population

Human serum samples were anonymously collected from January to the end of December 2020 in Siena as residual samples for unknown diagnostic purposes and stored at the laboratory of Molecular Epidemiology of the University of Siena, Italy, in compliance with Italian ethics laws. For each sample, only the information on the age, sex, and date of the collection were recorded.

A sample size per time period was established assuming a precision of the estimate of 2% with a confidence interval of 95% (95% CI) and an overall SARS-CoV-2 antibody prevalence of 2.96% [9].

A total of 2480 human serum samples were selected and stratified by time period according to the different first cases identified in Italy and the phases corresponding with the restrictive measures declared by the Italian government [3]. The time periods were indicated as follows: pre-lockdown phase (from 28 January to 8 March 2020); lockdown Phase 1 (from 9 March to 3 May 2020); Phase 2 (from 4 May to 14 June 2020); Phase 3 (subdivided into 3A from 15 June to 31 August 2020 and 3B from 1 September to 5 November 2020); and area-specific policies (from 6 November to 31 December 2020) (Table 1). The median age of the study population was 46.0 years with a range of 3–102 years; 1385 (55.85%) samples were from female subjects and 1095 (44.15%) were from males. Within the time period, the samples were stratified by sex and age group (0–46 and >46 years).

### 2.2. Serological Assays

#### 2.2.1. ELISA

The samples were tested by a commercial ELISA (Enzywell SARS-CoV-2 IgM and IgG, DIESSE, Siena, Italy) for the detection of IgM and IgG antibodies against SARS-CoV-2 by the use of ELISA plates coated with an inactivated whole-virus SARS-CoV-2 native antigen obtained from Vero E6 cells infected with SARS-CoV-2 [10,11]. The manufacturer, DIESSE, ensures a 92.5% sensitivity and 95.8% specificity for the IgG ELISA and 87.7% sensitivity and 97.0% specificity for the IgM ELISA. According to the manufacturer’s instructions, the samples were considered to be positive when the ratio between the optical density (OD) of the sample and that of the cut-off was >1.1, negative if the ratio was <0.9, and borderline if the ratio was between 0.9 and 1.1.

The samples with borderline or positive results for IgG and/or IgM were further tested by an in-house ELISA for the detection of IgG and IgM against the receptor-binding domain (RBD) of the spike (S) protein and by a micro-neutralization (MN) assay for the detection of a neutralizing antibody.

#### 2.2.2. In-House ELISA

The in-house ELISA was performed as previously reported [12]. Briefly, ELISA plates (Nunc, Maxi-Sorp) were coated with 1µg/mL of purified recombinant spike-RBD HEK-derived protein (Sino Biological, China). The human serum samples were diluted at a ratio of 1:100 in Tris Buffered Saline (TBS) 0.05% Tween 20 and 5% Non-Fat Dry Milk (NFDM, Euroclone, Pero, Italy) and then 100 µL of each serum dilution was added to the coated plates and incubated for 1 h at 37 °C. After the washing step, a goat anti-Human IgG-Fc or IgM μ-chain HRP-conjugated antibody (Bethyl Laboratories, Montgomery, TX, USA) was added and the plates were incubated at 37 °C for 30 min. After the washing step, a 3,3′,5,5′-Tetramethylbenzidine (TMB) substrate (Bethyl Laboratories, Montgomery, TX, USA) was added and incubated in the dark at room temperature for 20 min. The reaction was stopped and read at 450 nm.

#### 2.2.3. Micro-Neutralization Assay

The MN assay was performed as previously reported [13], using a wild-type SARS-CoV-2 (2019-nCov/Italy-INMI1 strain) virus purchased from the European Virus Archive Goes Global (EVAg, Spallanzani Institute, Rome, Italy). Briefly, the serum samples were heat-inactivated for 30 min at 56 °C and 2-fold serially diluted (starting dilution 1:10) then mixed with an equal volume of a SARS-CoV-2 viral solution containing 100 Tissue Culture Infective Dose 50% (TCID_50_). After 1 h of incubation at room temperature, 100 µL of each virus–serum mixture was added to a 96-well plate containing an 80% confluent Vero E6 cell monolayer. The plates were incubated for 3 days at 37 °C and 5% CO_2_ in a humidified atmosphere, then inspected for the presence/absence of a cytopathic effect (CPE) by means of an inverted optical microscope. The highest sample dilution showing no signs of a CPE was regarded as the neutralization titer.

### 2.3. Statistical Analysis

The categorical dichotomous data (sex), ordinal data (age group converted to a novel dummy variable comprising 0–46 and >46 years on the basis of the median age of the study population), and discrete data (commercial and in-house ELISA and MN assay results), defined as new categorical dichotomous variables, were described as counts and percentages and evaluated by a chi-squared test. The relations between the positivity of each IgM and IgG assay for each time period as a dependent categorical dichotomous variable and independent factors (sex and age group) were evaluated by a logistic regression model and the odds ratio (OR), 95% CI, and *p*-values were assessed. In the univariate logistic regression model, all the factors related to IgM and IgG positivity were investigated as independent variables. The statistically significant independent variables were assessed in the multivariate logistic regression model using a Wald test and a stepwise method for the selection of the *p*-value. The statistical analyses were performed using the online software package EZR, version 1.40 (Saitama Medical Centre, Jichi Medical University; Kanda, 2013) [14]. A *p* < 0.05 was considered to be statistically significant.

## 3. Results

### 3.1. Seroprevalence Rates of IgG and IgM Antibodies by the Commercial ELISA

The IgG and IgM results from the commercial ELISAs at different time periods of collection by sex and age group are reported in Table 2. Overall, of the 2480 samples collected throughout the study period, 133 (5.4%, 95% CI 4.5–6.3) were found to be positive or borderline to at least one antibody class. Positive or borderline results were found in 81 samples for IgG (3.3%, 95% CI 2.6–4.0) and in 58 samples for IgM (2.3%, 95% CI 1.8–3.0).

In the univariate logistic regression model, a statistical significance was observed throughout the study period between the positive and borderline results for IgG and age group and between the positive and borderline results for IgM and sex and age group. IgG positivity was statistically associated with age group (*p* = 0.001) with an OR of 2.23 (95% CI 1.37–3.61) whilst no association was observed with sex (*p* = 0.96). Positive results for IgM were statistically associated with sex (*p* = 0.005) with an OR of 0.43 (95% CI 0.24–0.78) and age group (*p* = 0.005) with an OR of 2.25 (95% CI 1.27–3.98). In the multivariate logistic regression model, the independent variables confirmed the statistical association between the positive or borderline results for IgG and IgM for each time period and in the entire study period.

**Figure 2:**
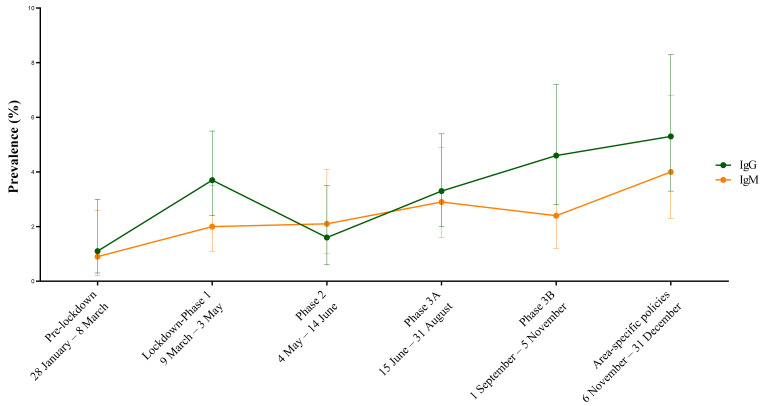
Prevalence over time periods by antibody class. Lines indicate IgG (green line) and IgM (yellow line) prevalence by commercial ELISA expressed as a percentage with 95%CI.

The seroprevalence trend over the time periods by the antibody class is shown in Figure 2.

Out of the 347 samples collected in the pre-lockdown period, 4 (1.1%, 95% CI 0.3–3.0) and 3 (0.9%, 95% CI 0.2–2.6) samples tested positive for IgG and IgM, respectively. Out of the 600 samples collected in Lockdown Phase 1, 22 (3.7%, 95% CI 2.4–5.5) and 12 (2.0%, 95% CI 1.1–3.5) samples tested positive or were borderline for IgG and IgM, respectively (Table 2). Out of the 382 samples collected in Phase 2, 6 (1.6%, 95% CI 0.6–3.5) and 8 (2.1%, 95% CI 1.0–4.1) samples tested positive or were borderline for IgG and IgM, respectively (Table 2). Out of the 455 samples collected in Phase 3A, 15 (3.3%, 95% CI 2.0–5.4) and 13 (2.9%, 95% CI 1.6–4.9) samples tested positive for IgG and IgM, respectively (Table 2). Out of the 373 samples collected in Phase 3B, 17 (4.6%, 95% CI 2.8–7.2) and 9 (2.4%, 95% CI 1.2–4.6) samples tested positive for IgG and IgM, respectively (Table 2). Out of the 323 samples collected in time period for area-specific policies, 17 (5.3%, 95% CI 3.3–8.3) and 13 (4.0%, 95% CI 2.3–6.8) samples tested positive or were borderline for IgG and IgM, respectively (Table 2).

In the univariate and multivariate logistic regression model, a consistent lack of association between the IgG and IgM results and the sex and age groups taken individually was observed for each time period. Conversely, positive IgG results were statistically associated with age group (*p* = 0.03, OR = 3.52, 95% CI 1.10–11.2) in Phase 3A and with sex (*p* = 0.01, OR = 3.56, 95% CI 1.28–9.90) in the time period for area-specific policies.

### 3.2. Seroprevalence of IgG and IgM Antibodies against RBD and Neutralizing Antibodies

The positive or borderline IgG and IgM samples obtained by the commercial ELISA were further tested by an RBD-based in-house ELISA and MN assay. The IgG and IgM results from the in-house ELISA and MN assay at different time periods of collection by sex and age group are reported in Table 3. Overall, 26 out of 81 (32.1%) and 11 out of 58 (18.9%) samples were found to be positive for RBD IgG and IgM, respectively. When tested by the MN assay, 23 out of 133 (17.3%) samples showed a neutralizing antibody (antibody titer range 10–1280). It was noteworthy that 27 out of 37 (72.9%) samples found to be positive for IgG and/or IgM against RBD showed neutralizing antibodies whereas all samples negative for RBD antibodies were also negative in the MN assay.

In the pre-lockdown period, one sample was positive for RBD IgG and a neutralizing antibody whilst no samples were positive for RBD IgM. During Lockdown Phase 1, one sample tested positive for RBD IgG whilst another sample was positive for RBD IgM; the latter was also positive for a neutralizing antibody. During Phase 2, one sample was positive for RBD IgG and a neutralizing antibody whilst no samples were found to be positive for RBD IgM. In Phase 3A, one sample was positive for RBD IgG and two samples were positive for IgM. The sample positive for RBD IgG was also positive for a neutralizing antibody (Table 3). In Phase 3B, nine samples were positive for RBD IgG and five were positive for IgM. Eight samples that tested positive for RBD IgG were also positive for a neutralizing antibody. In the time period for area-specific policies, 13 samples collected were positive for RBD IgG and 3 were positive for IgM; 11 samples that tested positive for RBD IgG were also positive for a neutralizing antibody.

We estimated the seroprevalence using a combination of the commercial ELISA and in-house ELISA/MN assay results. The total prevalence was 1.0% (95% CI 0.7–1.5) for RBD IgG, 0.4% (95% CI 0.2–0.8) for RBD IgM, and 0.9% (95% CI 0.6–1.4) for neutralizing antibodies.

**Figure 3:**
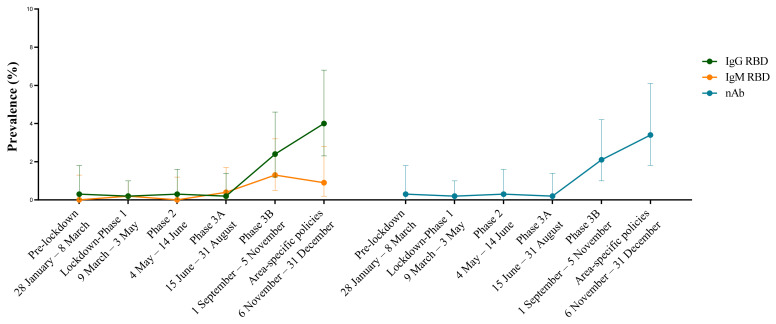
Prevalence over time periods by antibody class. On the left, the lines indicate IgG (green line) and IgM (yellow line) prevalence by in-house RBD ELISA. On the right, the blue line indicates neutralizing antibody prevalence by a micro-neutralization assay. Prevalence rates are expressed as percentages with 95% CI. nAb: neutralizing antibody.

The seroprevalence trend over the time periods by the RBD antibody class and neutralizing antibody is shown in Figure 3.

The first positive sample for IgG as well as for IgG RBD and a neutralizing antibody was collected on 2 March 2020. The prevalence of positive IgG RBD samples overlapped with the prevalence of a neutralizing antibody during all study periods, including the increase observed between Phase 3A and the time period for area-specific policies.

## 4. Discussion

In this study, the SARS-CoV-2 antibody prevalence in the population in the province of Siena in the Tuscany region of Italy from late January to December 2020 and prior to the general population vaccine deployment is presented. Overall, 5.4% of the samples had commercial IgG and/or IgM antibodies against SARS-CoV-2 in the time period under study.

The first positive sample for IgG as well as for IgG RBD and a neutralizing antibody was collected on 2 March 2020, 4 days after the first case of infection was detected in Siena (27 February) and 7 days before the national lockdown was implemented on 9 March. Considering that the median time to develop IgG antibodies has been estimated to be 14 days [15] after exposure, our results suggest that SARS-CoV-2 was circulating in the area well before the first case was ascertained, as reported from other studies [16,17,18].

The findings of this study show that the seroprevalence for SARS-CoV-2 remained very low in the Siena area until the end of August 2020 when a steady increase was observed until the end of the year. The data obtained from the surveillance system showed that as of 4 May 2020 (the first day of Phase 2), the number of positive cases registered in the province of Siena from the start of the pandemic was 425. During the second wave, the incidence of new cases in the province of Siena reached 318 new positive cases per 200,000 inhabitants. As of 13 November 2020, when the Tuscany region was declared a “red zone”, the number of positive cases in the province of Siena was 3160 and reached 4959 cases at the end of the year. Thus, the seroprevalence trend observed in this study was in line with the epidemiological data.

Seroprevalence studies conducted in Italy during and immediately after the first epidemic wave reported values ranging from 2.6% to 22.6% [19,20,21,22,23]. A study conducted in another province of the Tuscany region [9] found a prevalence of 2.96% and between May and July 2020, the Italian National Institute of Statistics (ISTAT) assessed a prevalence of 1% in the Tuscany region [24].

A trend toward an increase was observed in late 2020, starting from the end of summer, which was consistent with the epidemiological trend in the region. The Tuscany region was affected by the SARS-CoV-2 pandemic mostly during the late summer–autumn season when the second and higher pandemic wave occurred in Italy. Despite this, the prevalence values were relatively low. The low prevalence may be explained by the implementation of extensive preventive measures on the population, especially during the first epidemic wave. Fiore et al. [25] highlighted that studies from Italy and other countries that adopted strict lockdown measures reported low prevalence values, comparable with those detected in countries that opted for a herd immunity strategy with fewer and lighter restrictions.

In this study, we observed that IgG and/or IgM positivity were found to be strongly associated with age with lower prevalence rates in older subjects, probably because of targeted efforts to reduce social interactions in this age group. The stronger social distancing combined with immunosenescence might have led to a lower prevalence, as previously suggested [26].

A SARS-CoV-2 infection in humans elicits a predominant antibody response, mainly targeting the S protein and, in particular, against RBD [15]. In this study, we used an RBD ELISA and MN as tools to characterize the immune response to SARS-CoV-2. A total of 71.9% of samples exhibiting antibodies against RBD were also able to neutralize the wild-type virus in the MN assay, supporting the fact that antibodies directed against RBD of the S protein are highly neutralizing [12].

This study has a few limitations. The use of residual samples may not be completely representative of the population. Subjects who did not undergo analytical testing during 2020 were not included in the sample collection. Moreover, a lack of information regarding the clinical manifestations and outcomes did not allow us to evaluate the proportion of asymptomatic and symptomatic infections, and no information was available on the recent travel or social contacts of the subjects. Samples were collected at a single center (Siena), which may have introduced a bias.

The ELISA could have exhibited a degree of cross-reactivity with antibodies to other human coronaviruses, leading to an overestimation of the actual seroprevalence due to false-positive results. In the context of low prevalence values such as those found in this study, the combination of more than one serological test provides a more reliable estimation of the real values. Finally, our results may represent an underestimation of the proportion of subjects who experienced a SARS-CoV-2 infection because not all infected subjects develop antibodies; antibody titers may be lower in mild cases and even undetectable with a commercial ELISA. A few may have lost antibodies or not yet developed antibodies after a recent infection [27,28,29,30].

A key strength of this study is that the presence of a neutralizing antibody was determined in vitro by using a live SARS-CoV-2 strain circulating in Italy in 2020. Such a seroprevalence study provides information not only about previous exposure to SARS-CoV-2, but also immunity to the virus.

To our knowledge, this is the first seroepidemiological study conducted in Italy to evaluate the status of immunity to SARS-CoV-2 in a sample population during the whole of 2020. This study provides important insights regarding the general population, given that the sample collection was performed before the start of vaccination campaigns and covers both the first and second waves of infection. Our results were consistent with the reports from other regions across the world, showing that only a minority of the population was infected with SARS-CoV-2 during the first year of the pandemic even in areas with widespread virus circulation [26,31,32,33]. Considering the high morbidity and mortality burden of COVID-19, the option of aiming to reach herd immunity in the general population as a consequence of exposure to a natural infection cannot be considered to be a viable option compared with vaccination to ensure immunity in the population.

## 5. Conclusions

In conclusion, in our study we showed the importance of serological studies as tools that can provide information on the extent of the circulation of a given pathogen in the population and the status of immunity, helping to adopt sound public health measures and to properly follow and evaluate their impact on pandemics.

## Figures and Tables

**Table 1:** Study population serum samples collected in Siena (Tuscany region, Central Italy) from January to December 2020 by time period.

	Time Period						Total
	Pre-Lockdown 28 January–8 March	Lockdown Phase 1 9 March–3 May	Phase 2 4 May–14 June	Phase 3A 15 June–31 August	Phase 3B 1 September–5 November	Area-Specific Policies 6 November–31 December	
Number of samples	347	600	382	455	373	323	2480

**Table 2:** Information of subjects (age group and sex) and serological results (commercial ELISA) of the serum samples collected at different time periods.

Time Period	Antibody	Result	Age Group		Sex		Total
			0–46	>46	M	F	
Pre-lockdown	IgG	P	3	1	1	3	4
		N	181	162	166	177	343
		B	0	0	0	0	0
		T	184	163	167	180	347
	IgM	P	3	0	0	3	3
		N	181	163	167	177	344
		B	0	0	0	0	0
		T	184	163	167	180	347
Lockdown Phase 1	IgG	P	13	8	9	12	21
		N	255	323	268	310	578
		B	0	1	0	1	1
		T	268	332	277	323	600
	IgM	P	6	3	2	7	9
		N	260	328	275	313	588
		B	2	1	0	3	3
		T	268	332	277	323	600
Phase 2	IgG	P	3	2	1	4	5
		N	189	187	195	181	376
		B	1	0	1	0	1
		T	193	189	197	185	382
	IgM	P	4	3	4	3	7
		N	188	186	192	182	374
		B	1	0	1	0	1
		T	193	189	197	185	382
Phase 3A	IgG	P	11	3	7	7	14
		N	193	247	199	241	440
		B	0	1	1	0	1
		T	204	251	207	248	455
	IgM	P	5	7	2	10	12
		N	199	243	204	238	442
		B	0	1	1	0	1
		T	204	251	207	248	455
Phase 3B	IgG	P	12	5	4	13	17
		N	218	138	128	228	356
		B	0	0	0	0	0
		T	230	143	132	241	373
	IgM	P	8	1	1	8	9
		N	222	142	131	233	364
		B	0	0	0	0	0
		T	230	143	132	241	373
Area-specific policies	IgG	P	14	3	11	6	17
		N	202	104	104	202	306
		B	0	0	0	0	0
		T	216	107	115	208	323
	IgM	P	11	1	4	8	12
		N	204	106	111	199	310
		B	1	1	0	1	1
		T	216	107	115	208	323
Total	IgG	P	56	22	33	45	78
		N	1238	1161	1060	1339	2399
		B	1	2	2	1	3
		T	1295	1185	1095	1385	2480
	IgM	P	37	15	13	39	52
		N	1254	1168	1080	1342	2422
		B	4	2	2	4	6
		T	1295	1185	1095	1385	2480

P: positive; N: negative; B: borderline; T: tested.

**Table 3:** Information of subjects (age group and sex) and serological results (in-house ELISA and micro-neutralization assay) of the serum samples collected at different time periods.

Time Period	Antibody	Result	Age Group		Sex		Total
			**0–46**	**>46**	**M**	**F**	
Pre-lockdown	RBD IgG	P	1	0	0	1	1
		N	2	1	1	2	3
		T	3	1	1	3	4
	RBD IgM	P	0	0	0	0	0
		N	3	0	0	3	3
		T	3	0	0	3	3
	nAb	P	1	0	0	1	1
		N	5	1	1	5	6
		T	6	1	1	6	7
Lockdown Phase 1	RBD IgG	P	0	1	0	1	1
		N	13	8	9	12	21
		T	13	9	9	13	22
	RBD IgM	P	1	0	1	0	1
		N	7	4	1	10	11
		T	8	4	2	10	12
	nAb	P	1	0	1	0	1
		N	20	13	10	23	33
		T	21	13	11	23	34
Phase 2	RBD IgG	P	1	0	0	1	1
		N	3	2	2	3	5
		T	4	2	2	4	6
	RBD IgM	P	0	0	0	0	0
		N	5	3	5	3	8
		T	5	3	5	3	8
	nAb	P	1	0	0	1	1
		N	8	4	6	6	12
		T	9	4	6	7	13
Phase 3A	RBD IgG	P	1	0	0	1	1
		N	10	4	8	6	14
		T	11	4	8	7	15
	RBD IgM	P	2	0	0	2	2
		N	3	8	3	8	11
		T	5	8	3	10	13
	nAb	P	1	0	0	1	1
		N	15	12	11	16	27
		T	16	12	11	17	28
Phase 3B	RBD IgG	P	8	1	2	7	9
		N	4	4	2	6	8
		T	12	5	4	13	17
	RBD IgM	P	5	0	0	5	5
		N	3	1	1	3	4
		T	8	1	1	8	9
	nAb	P	7	1	2	6	8
		N	11	5	3	13	16
		T	18	6	5	19	24
Area-specific policies	RBD IgG	P	10	3	11	2	13
		N	4	0	0	4	4
		T	14	3	11	6	17
	RBD IgM	P	3	0	3	0	3
		N	9	1	1	9	10
		T	12	1	4	9	13
	nAb	P	8	3	10	1	11
		N	15	1	2	14	16
		T	23	4	12	15	27
Total	RBD IgG	P	21	5	13	13	26
		N	36	19	22	33	55
		T	57	24	35	46	81
	RBD IgM	P	11	0	4	7	11
		N	30	17	11	36	47
		T	41	17	15	43	58
	nAb	P	19	4	13	10	23
		N	74	36	33	77	110
		T	93	40	46	87	133

P: positive; N: negative; T: tested; nAb: neutralizing antibody.

## Data Availability

Data are contained within the article.
